# Notes and News

**Published:** 1901-09-07

**Authors:** 


### Notes and News.

The experiments in Bellinzaghi's yellow-fever serum have not proved very encouraging. Two Spaniards, who offered themselves for experiment in Havana after being bitten by mosquitoes, both died, in spite of the use of the serum.

Mr. Andrew Barlow, of Shirley, Southampton, has made a donation of £4,500 to the Royal South Hants Infirmary, of £1,700 to the Southampton Dispensary, of £1,500 to the Southampton Free Eye Hospital, and several other large sums to different charities. Mr. Barlow is well known for his generosity in the district.

The new law relating to the standard of milk is now in operation. The standard now fixed requires that milk shall contain not less than 3 per cent. of fat and not less than 8·5 per cent. of milk solids other than fat. Below this standard, which cannot be called a high one, it is now illegal to sell milk. Previously local public analysts were left to form their own judgment as to a fitting standard.

The New Orleans Board of Health has commenced a crusade against mosquitoes, and two wards of the city have been placed under special sanitary inspectors, who have visited all the premises under their supervision. Paraffin has been chosen as the agent wherewith to combat the mosquitoes. Crude Beamont oil is to be used in the gutters and open ponds, and paraffin in the cisterns. The experiment cannot fail to be an interesting one and may prove very instructive generally.

During the present holiday season the presence of an unusual amount of smallpox will have passed almost unnoticed by the majority of Londoners. The epidemic which has been mainly confined to the districts round St. Pancras and St. Marylebone, has been combated in a very efficient manner. The notification has been very thorough and the ambulance system of the Metropolitan Asylums Board very prompt and effective. Cases have been speedily removed to the hospital ships at Long Reach, Dartford. Here the increase of work has been felt and steps taken to add to the nursing staff. The total number of patients under treatment is eighty-four.

The Soldiers' Convalescent Home at Fairfield is about to be closed, owing to the unsatisfactory conduct of the inmates. It was not very difficult to predict that small institutions improvised and supported by the lay public were not likely to be very satisfactory for military men, even if they were useful. Much of the money collected for the purpose has never been utilised at all. There are many other more practicable and useful ways of helping soldiers who have come out of hospital, which still remain for the generous to take advantage of.

Major Ross has sent to the Liverpool School of Tropical Medicine a most interesting account of his visit to Lagos. Major Ross shows that the inhabitants of Lagos are themselves carrying on a systematic campaign against malaria, in which the Governor, Sir William MacGregor, and the Ladies' League take a prominent part. The marshes in the district are being drained and filled with sand, numbers of houses have been rendered mosquito-proof by fine wire netting, and practical knowledge in sanitary matters is disseminated amongst the people.

It is not often nowadays that the old terror of hospitals is actively demonstrated, but that it is not wholly dead is shown by the action of a mother living in the West End whose daughter contracted smallpox. The case was notified, and the dwelling proving wholly unsuitable for home treatment, an order for removal to the Asylums Board Hospital was made. The mother, however, refused to permit the child to be removed, stating her belief that she would be either poisoned or killed. An application was made to the courts for an order for forcible removal, which was granted. Then another difficulty arose. The warrant officer did not want to be exposed to infection unnecessarily, and the magistrate expressed a doubt whether a recently re-vaccinated police-constable was available. The Holborn Town Clerk pointed out this was a serious matter, in view of the present prevalence of smallpox.

At the last quarterly court of governors of the Middlesex Hospital, it was stated that the special grant from the Prince of Wales's Hospital Fund of £250 made to the hospital in December last had been returned. The grant was made conditionally that the hospital should supply free to all patients tea, sugar, and butter, and provide for certain clothing and washing, conditions which the board were unable to accept, owing to the additional expense which would in consequence fall on the hospital funds, and which would greatly exceed the amount of the grant in question. A suggestion by the board that the grant should be made to the Samaritan Fund of the hospital, which is laid under contribution continually to a considerable extent for the provision of such articles as tea, sugar, and butter to all impecunious patients, was unfavourably received by the committee of the Prince of Wales's Fund. It was announced that Mr. T. H. Kellock, F.R.C.S., will deliver the introductory address to the students at the opening of the winter session of the medical school on October 1st, and Mr. John Bland-Sutton, F.R.C.S., will preside at the annual dinner to be held the same evening at the Trocadero Restaurant. The expenditure of £11,500 on a laundry at Hendon was sanctioned, and a pavilion for the open-air treatment of tuberculosis, to accommodate four male patients, is to be built in the grounds of the convalescent home at Clacton-on-Sea.

